# Shear stress-induced mechanotransduction protein deregulation and vasculopathy in a mouse model of progeria

**DOI:** 10.1186/scrt429

**Published:** 2014-03-24

**Authors:** Minjung Song, Hong San, Stasia A Anderson, Richard O Cannon III, Donald Orlic

**Affiliations:** 1 Translational Medicine Branch, National Heart, Lung, and Blood Institute, National Institutes of Health, Bethesda, MD, USA; 2 Genome Technology Branch, National Human Genome Research Institute, National Institutes of Health, Bethesda, MD, USA

## Abstract

**Introduction:**

A mouse model of progeria derived by insertion of the human mutant *LMNA* gene (*mLMNA*), producing mutant lamin A, shows loss of smooth muscle cells in the media of the ascending aorta. We hypothesized that high shear stress, in the presence of mutant lamin A, induces this vasculopathy and tried to define the molecular and cellular basis for aortic vasculopathy.

**Methods:**

Ascending and descending aortas from wild type (WT) and mLMNA^+^ mice were compared using proteomics, Western blots, PCR and immunostaining. To determine whether high fluidic shear stress, known to occur in the ascending aorta, contributed to the vasculopathy, we exposed descending aortas of mLMNA^+^ mice, with no apparent vasculopathy, to 75 dynes/cm^2^ shear stress for 30 minutes using a microfluidic system.

**Results:**

When the mice were one year of age, expression of several mechanotransduction proteins in the ascending aorta, including vimentin, decreased in mLMNA^+^ mice but no decrease occurred in the descending aorta. High fluidic shear stress produced a significant reduction in vimentin of mLMNA^+^ mice but not in similarly treated WT mice.

**Conclusions:**

The occurrence of mutant lamin A and high shear stress correlate with a reduction in the level of mechanotransduction proteins in smooth muscle cells of the media. Reduction of these proteins may contribute over time to development of vasculopathy in the ascending aorta in progeria syndrome.

## Introduction

Hutchinson-Gilford Progeria Syndrome (HGPS) is an autosomal dominant genetic disease that involves accelerated aging in children, leading to early death due to myocardial infarction or stroke [1]. Autopsies indicate the principle phenotypic defect occurs in the media of large conducting arteries, including the aortas and carotids, with a loss of smooth muscle cells (SMCs) and extensive accumulation of extracellular matrix.

Lamin A, a product of the *LMNA* gene, is a nuclear protein involved in structural support, DNA replication and gene transcription through protein-protein interactions [2-7]. The progeria syndrome occurs as a result of a point mutation at G608G of the *LMNA* gene. Substitution of C for T at nucleotide 1824 activates a cryptic splice donor site in exon 11 that results in a 50-amino acid deletion near the C terminus. Consequently, the protein cannot undergo the final proteolytic processing step in the lamin A maturation pathway and thus is modified by retention of a lipid farnesyl group. This mutant form of lamin A, called progerin, is permanently anchored at the nuclear envelope [8,9].

An experimental mouse model of progeria was generated by insertion of the human mutant lamin A gene (*mLMNA*) using a bacterial artificial chromosome [9]. As *mLMNA*^*+*^ mice age, loss of SMCs occurs in the media of the aortas and carotid arteries, with accumulation of matrix proteoglycan [8]. These changes resemble those reported for the aorta and carotid arteries in HGPS patients [1,10].

Although the mutant gene product, progerin, is present in both the ascending and descending aortas in this animal model, we observed vasculopathy only in the ascending aortas of 12-month-old mice. Interestingly, the ascending aorta is subjected to the highest fluidic shear stress during each cardiac systole [11-13], with the highest shear stress occurring in the media compared with the intima and adventitia across the aortic wall [11].

We proposed that progerin in SMCs of the media alters their normal adaptation to high shear stress through deleterious effects on gene expression and/or protein conservation. Using wild type (WT) and *mLMNA*^*+*^ mice, we performed proteomic, molecular, immunohistochemical and fluidic shear stress analyses, and determined that mechanotransduction proteins, including vinculin, transgelin and vimentin, all type III intermediate filaments, may be key proteins that contribute to the vasculopathy seen in the progeria syndrome.

## Materials and methods

### Progeria mouse model

A mouse model of HGPS was generated using a bacterial artificial chromosome (BAC) in the laboratory of Dr. Francis S. Collins [8]. In brief, the BAC was engineered to carry the single nucleotide mutation at G608G of the human lamin A gene coding for the dysfunctional protein, progerin, which is responsible for the progeria syndrome in children [8]. All mice were genotyped using primers 5′-GTGTCTGGGTGCCCTACTCTGGTAAGGA-3′ and 5′-GTAGGGCAGCAGGCATGCACTATTA-3′. The investigation conforms to the *Guide for the Care and Use of Laboratory Animals* published by the US National Institutes of Health (NIH Publication No. 85–23, revised 1996). All animals were handled following the guidelines of the Animal Care and Use Committee at the National Institutes of Health. The animal study protocol was approved by the Animal Care and Use Committee of the NIH (Animal Study H-0165) and the approved protocol affected all the centers involved in the current study.

### Magnetic resonance imaging

Mice were anesthetized with 1 to 2% isoflurane and imaged with electrocardiography (ECG), respiratory and temperature detection using a 7.0 T, 160 mm horizontal Bruker magnetic resonance imaging (MRI) system (Bruker, Billerica, MA, USA) equipped with a 38 mm birdcage volume coil. Magnevist (Bayer HealthCare, Montville, NJ, USA) diluted 1:10 with sterile 0.9% phosphate-buffered saline, was administered subcutaneously or intravenously via tail vein at a dose of 0.3 mmol/L gadolinium/kg. Two-dimensional multi-slice spin echo images of the aortas were acquired across the aortic root with ECG and respiratory gating with the following parameters: TR/TE = 700/10.8 ms, three to four averages, 0.5 to 0.6 mm slice thickness, 2.5 to 2.7 cm field of view, 256 × 256 matrix, resolution approximately 100 × 100 × 500 μm^3^. For mice that were also evaluated for left ventricular function, gradient echo cine images were acquired in short axis from base to apex, with ECG and respiratory gating. Imaging parameters were TR/TE = 12 to 13/3.2, 30 degree flip angle, 11 to 12 frames, four averages, 1.0 mm slice thickness, 28 mm field of view, 256 × 256 matrix, resolution approximately 110 × 110 × 1,000 μm^3^. Cardiac MRI data were processed using CAAS-MRV-FARM software (Pie Medical Imaging, Maastricht, The Netherlands).

### Proteomic analysis

Ascending and descending aortas were homogenized for 10 minutes in protein lysis buffer and a methanol-cleaning step followed to remove adipose tissues. Protein concentrations were determined using the 2-D Quant kit (GE Healthcare, Piscataway, NJ, USA). Differences were obtained by gel electrophoresis on triplicate samples. Specifically, 50 μg of aorta protein from WT and *mLMNA*^*+*^ mice were labeled with Cy3 and Cy5 dyes (GE Healthcare). A standard protein solution containing 25 μg of each sample was labeled with Cy2. Labeled Cy2-, Cy3- and Cy5- samples were loaded on an immobilized pH gradient strip (pH 3 to 10, GE Healthcare). The first-dimension electrophoresis was run on an Ettan IPGphor IEF system. Then, the IPG strips were equilibrated and second-dimension separation was performed in 10 to 15% gradient gels using an Ettan DALTSix™ electrophoresis (GE Healthcare).

Gels were scanned on a Typhoon 9400 scanner (GE Healthcare) at 100 μm resolution. The images were processed with Progenesis Discovery software (Nonlinear Dynamics, Newcastle, UK). The software quantifies the ratio of Cy3 WT and Cy5 *mLMNA*^*+*^ spot intensities and statistical significance was determined by a two-sided Student’s *t*-test (n = 3). Spots with greater than a 1.6-fold change were selected for identification.

The Ettan Spot Handling Workstation was used to extract the peptides from the gel and transfer the digested proteins on a matrix-assisted laser desorption/ionization time of flight mass spectrometry (MALDI-TOF/Pro) (GE Healthcare). Peptides were analyzed using a 4700 Proteomics Analyzer (Applied Biosystems, Foster City, CA, USA) tandem MS/MS and proteins were identified with the MASCOT database search function.

### Western blot

Proteins from each sample were applied to a 10% tris-glycine gel (Invitrogen, Carlsbad, CA, USA) and incubated with primary antibodies overnight at 4°C. Primary antibodies included rabbit anti-transgelin (1:1,000), mouse anti-vinculin (1:200), rabbit anti-vimentin (1:500), (Abcam, Cambridge, MA, USA) and mouse anti-glyceraldehyde 3-phosphate dehydrogenase (GAPDH) (1:5,000, Ambion, Austin, TX, USA). Secondary antibodies were horseradish peroxidase-conjugated goat anti-rabbit or anti-mouse antibodies (Pierce, Rockford, IL, USA). Enhanced chemiluminescence reaction was performed using a SuperSignal® West Femto enhancer kit (Pierce).

### Immunohistochemistry and confocal microscopy

Aortas were fixed in 4% formaldehyde solution and 5 μm thick paraffin embedded sections were prepared (HistoServ, Germantown, MD, USA). After rehydration, sections were incubated with primary antibody at 4°C overnight in a moist chamber followed by secondary antibody incubation for one hour at 37°C. Primary antibodies included mouse anti-smooth muscle α-actin (ab5694, Abcam), rabbit anti-vimentin (ab45939, Abcam) and mouse anti-human lamin A/C (Millipore, Palm Springs, CA, USA). Secondary antibodies included donkey anti-rabbit, donkey anti-mouse and donkey anti-goat fluorescein isothiocyanate (FITC) and donkey anti-rabbit Texas Red® from Jackson Immuno Research (West Grove, PA, USA). The sections were mounted with Vectashield containing 4′, 6-diamidino-2-phenylindole (DAPI) to detect cell nuclei. Images were obtained with a confocal microscope (Leica SP1, Leica Microsystems, Heidelberg, Germany) and Leica confocal software.

### Shear stress experiment

Fluidic shear stress was applied to the ascending and descending aortas of 12-month-old WT and *mLMNA*^*+*^ mice. A flow system was set up to impose artificial shear stress on the ascending and descending aortas at 75 dynes/cm^2^ for 30 minutes by controlling media flow rate. This matched the highest level observed in the mouse cardiovascular system [14]. Wall shear stress (τ) was calculated using the formula: τ = 4ηQ/πr^3^, where η is the viscosity of the media and r is vessel radius. Tubes were connected to a microfluidic chamber (Harvard Apparatus, Holliston, MA, USA) and catheters were inserted into the origin of the ascending aortas. Dulbecco’s Modified Eagle Medium (Invitrogen) flowed through the ascending and descending aortas and exited at the vena cava. An additional image file shows this in more detail (see Additional file 1).

### RNA isolation and RT-PCR

To measure vimentin mRNA levels, RT-PCR was performed on the aortas of *mLMNA*^*+*^ mice before and after shear stress. We carefully removed as much of the adventitial layer as possible using a high power dissection microscope, and subsequently harvested the descending aortas. These consisted mostly of media with a single cell layer of endothelium. Total RNA was isolated from the descending aortas using the RNeasy mini kit (Qiagen, Valencia, CA, USA) and reverse transcriptase-polymerase chain reaction (RT-PCR) was carried out with a SuperScript kit (Invitrogen). The vimentin primer was purchased from Qiagen. The PCR products were analyzed by agarose gel electrophoresis. Beta-actin was used as an internal control.

## Results

### Pathologic phenotypes of *mLMNA*^*+*^ mice

**Figure 1:**
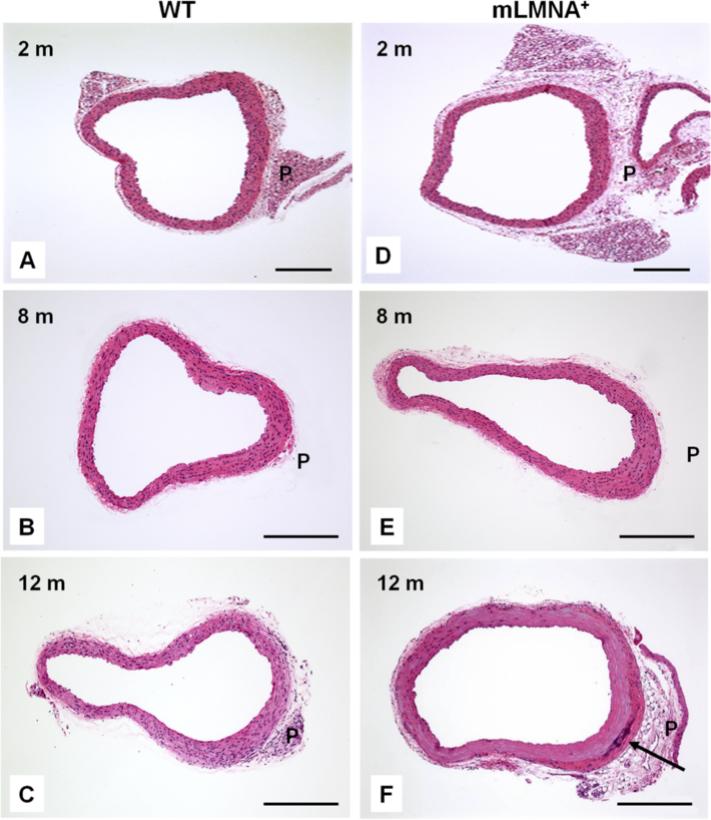
**Histologic analysis of cross sectional images of the ascending aorta in WT and *mLMNA***^***+***^**mice.** In this series of hematoxylin and eosin stained slides, changes are observed in the ascending aorta in wild type (WT) **(A-C)** and *mLMNA*^*+*^**(D-F)** mice during aging. Prominent thickening of the media occurs in the posterior wall (P) of WT mice but the media remains intact with a complete layer of smooth muscle cells (SMCs). In contrast, progressing at 12 months (F), the thickened media in the posterior wall of *mLMNA*^*+*^ mice becomes largely depleted of SMCs and extensive extracellular matrix is deposited. These damaged areas are bounded by a prominent adventitia that consists of fibroblasts, a dense layer of collagen and calcification at the boundary of media and adventitia (arrow) (m = months). Scale bar: 250 μm.

A series of hematoxylin and eosin stained preparations show changes in the ascending aorta in WT (Figure 1A-C) and *mLMNA*^*+*^ (Figure 1D-F) mice over a period of 2 to 12 months after birth. In cross-sectional views of the ascending aortas at all time-intervals, the endothelial lining of the intima remains intact as a single layer of endothelial cells. A thickening of the posterior wall, noted at 2 to 6 months, became prominent at 8 to 12 months in both WT and *mLMNA*^*+*^ mice. The aorta in WT mice shows a thickening of the posterior media similar to that seen in *mLMNA*^*+*^ mice, but there is no apparent loss of SMCs or change in the adventitial layer. In contrast, the thickened media of the posterior wall of *mLMNA*^*+*^ mice shows extensive loss of SMCs at 12 months, with replacement by extracellular matrix (Figure 1F). An additional difference was noted at 12 months, the damaged media in *mLMNA*^*+*^ mice is bordered by an expanded adventitia consisting of fibroblasts, a dense layer of collagen and focal calcification (Figure 1F). These structural changes in the ascending aorta of *mLMNA*^*+*^ mice do not occur in the descending aorta of *mLMNA*^*+*^ mice at this age.

**Figure 2:**
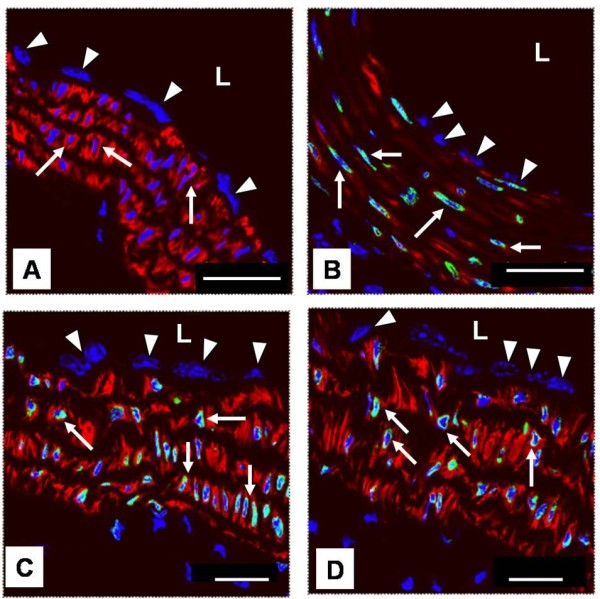
**Confocal microscopy of progerin expression in 12-month-old WT and *mLMNA***^***+***^**mice.** In the ascending aortas in wild type (WT) mice **(A)**, nuclei of endothelial cells (arrowheads) and smooth muscle cells (SMCs) (arrows), as expected, are negative for progerin expression. In *mLMNA*^*+*^ mice **(B),** the endothelial cells (arrowheads) in the ascending aortas are progerin negative, whereas all SMCs (arrows) in the intact region of the media show progerin deposition in their nuclei, mostly at the peripheral margin. A similar pattern of progerin expression is seen in the descending segment of the aortas in *mLMNA*^*+*^ mice **(C and D)**; all SMC nuclei (arrows) are progerin positive, but the endothelial cells are negative (arrowheads). The lumen of each vessel is labeled (L). Blue = DAPI, green = progerin, red = SMαA. Scale bar: 25 μm.

Progerin expression in the ascending and descending aortas and organs of 12-month-old *mLMNA*^*+*^ mice was assessed by immunostaining and confocal microscopy. As expected, all cells were negative for progerin expression in the aortas (Figure 2A) and other organs in WT control mice. Surviving SMCs in the media of the ascending aortas of *mLMNA*^*+*^ mice and in the intact descending aortas of these mice showed extensive progerin deposition at the peripheral margin of nuclei (Figure 2B-D). Unexpectedly, progerin was not detected in nuclei of endothelial cells in the ascending aortas (Figure 2B) or descending aortas (Figure 2C, D).

### MRI analysis of the lumen of the aortic root

**Figure 3:**
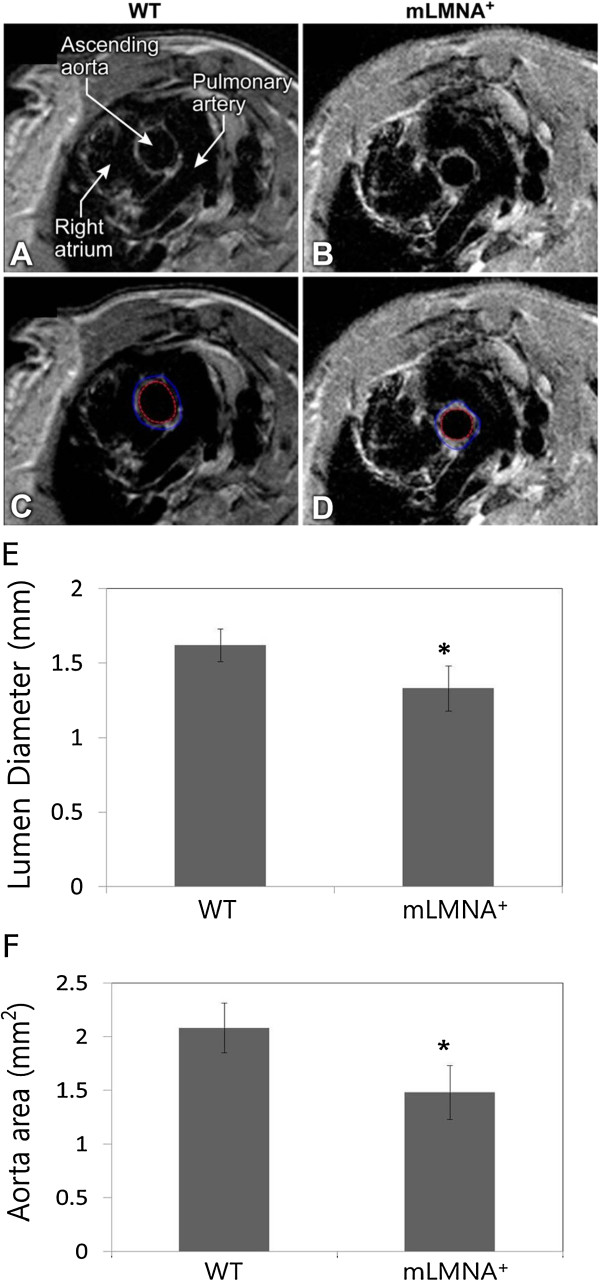
**MRI of the aorta.** Cross sections of the origin of the ascending aortas seen by spin-echo magnetic resonance imaging (MRI) in a wild type **(A)** and an *mLMNA +* **(B)** mouse. Images include the ascending aorta in cross-section, the right atrium and the pulmonary artery, annotated on image (A). The measured area for aortic wall thickness and lumen cross sectional area are shown in the bottom row with two regions of interest (ROIs) indicating the inner and outer borders of the aortic wall in the wild type (WT) **(C)** and *mLMNA*^*+*^ mouse **(D)**. The lumenal cross section is the area of the inner region of interest (ROI) multiplied by the slice thickness; the wall area is the area between the two ROI outlines multiplied by the slice thickness. Lumen diameter (mm) **(E)** and aorta area (mm^2^) **(F)** of wild type and *mLMNA*^*+*^ mice (n = 3).

We measured the lumen diameter during diastole at the root of the ascending aortas in 12-month-old mice using MRI (Figure 3A-D) and found a significant (*P* <0.05) reduction in *mLMNA*^*+*^ (n = 3) mice (1.33 ± 0.15 mm, n = 7) compared with WT (n = 3) mice (1.62 ± 0.10 mm, n = 4) (Figure 3E). Likewise, the average aorta area was reduced in *mLMNA*^*+*^ mice (Figure 3F). This reduction in the diameter of the lumen and aorta area may lead to an increase in mechanical stress and thus account for some of the vasculopathy observed in the medial wall of the ascending aortas in *mLMNA*^*+*^ mice.

### Protein profiles in WT and *mLMNA*^*+*^ ascending aortas

**Figure 4:**
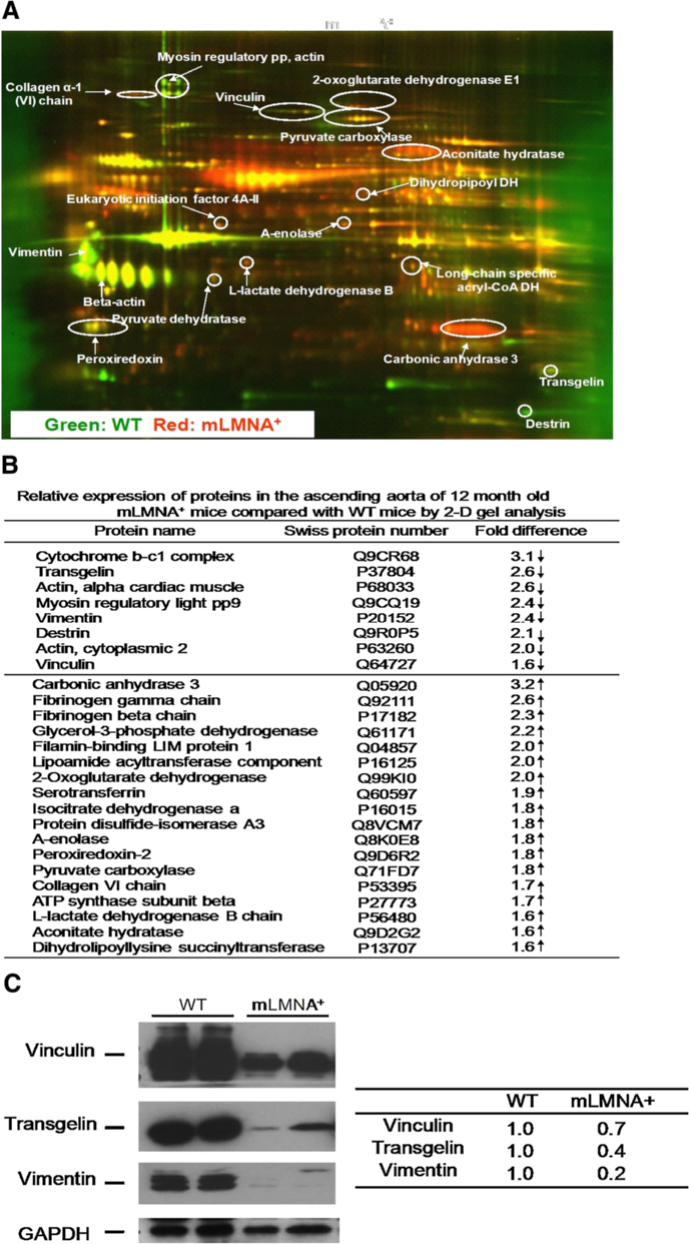
**Proteomic analysis and mechanotransduction protein expression of ascending aortas in WT and *mLMNA***^***+***^**mice. (A)**: Proteomic analysis of the ascending aortas in wild type (WT) and *mLMNA*^*+*^ mice. This two-dimensional (2-D) gel image shows protein expression in the ascending aortas of WT (green) and *mLMNA*^*+*^ (red) mice at 12 months. Yellow spots indicate commonly expressed proteins. The image suggests relatively greater expression of cytoskeleton (myosin regulatory light polypeptide 9, destrin, cytochrome b-c1 complex, actins) and mechanotransduction-related (vimentin, vinculin, transgelin) proteins in WT relative to *mLMNA*^*+*^ mice, and relatively greater expression in mitochondrial enzymes and extracellular matrix components in *mLMNA*^*+*^ mice relative to WT mice. A complete list of proteins can be found in **(B)**. **(C)**: Mechanotransduction protein levels in the ascending aorta of 12-month-old WT and *mLMNA*^*+*^ mice. When vinculin, transgelin and vimentin bands were normalized to glyceraldehyde 3-phosphate dehydrogenase (GAPDH), the quantity of each protein was reduced in *LMNA*^*+*^ mice compared with WT. The level of vimentin was the most significantly reduced.

We tested the hypothesis that progerin modifies gene expression in the ascending aortas by comparing the protein profiles of WT and *mLMNA*^*+*^ mice using proteomic analysis (Figure 4A). Proteins from WT and *mLMNA*^*+*^ mice were conjugated with Cy3 (green) and Cy5 (red), respectively. In two-dimensional (2-D) gels, a large number of differentially expressed proteins were observed in WT and *mLMNA*^*+*^ ascending aortas. Using MALDI-TOF analysis, approximately 26 proteins showed a greater than 1.6-fold difference between WT and *mLMNA*^*+*^ aortas. Eight proteins were down-regulated in the *mLMNA*^*+*^ ascending aortas (Figure 4B). Most down-regulated proteins were cytoskeleton-related or mechanotransduction-related. Eighteen up-regulated proteins in the *mLMNA*^*+*^ ascending aortas included many mitochondrial enzymes and the extracellular matrix protein, collagen VI.

Among the down-regulated proteins, we focused on those with mechanotransduction properties that might be responsive to the high shear stress encountered in the ascending aortas [15-17]. At 12 months, vinculin, transgelin and vimentin were highly expressed in the WT group as seen by Western blot, whereas levels of expression were reduced in *mLMNA*^*+*^ mice (Figure 4C). Based on triplicate samples, vimentin expression, normalized to GAPDH, was five-fold greater in the ascending aortas of WT mice compared with *mLMNA*^*+*^ mice at 12 months.

### Vimentin expression in the intima, media and adventitia

**Figure 5:**
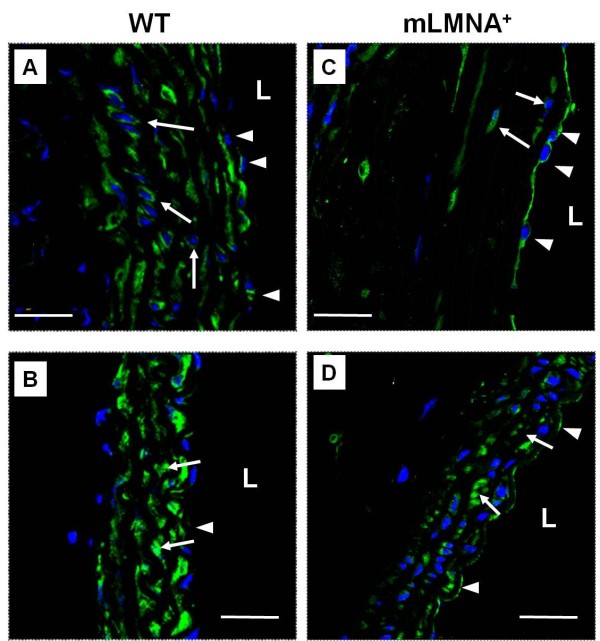
**Immunostaining of vimentin in the ascending aortas in WT and *mLMNA***^**+**^** mice at 12 months.** The pattern of vimentin expression in the ascending aortas in wild type (WT) mice **(A and B)** was compared with *mLMNA*^*+*^ mice **(C and D)** by immunostaining and confocal microscopy. In WT mice (A) the media of the posterior wall is cellular and vimentin positive (arrows), whereas in *mLMNA*^*+*^ mice (C) the media of the posterior wall is mostly acellular, but the few surviving SMCs show vimentin staining (arrows). All endothelial cells (arrowheads) are vimentin positive (A and C). Cells of the intima and media in the descending aortas in 12-month-old WT (A) and *mLMNA*^*+*^ (B) mice were analyzed for vimentin expression. The contiguous monolayer of vimentin positive endothelial cells in each vessel is indicated (arrowheads) as is the SMC layers of the media (arrows). The lumen of each vessel is indicated (L). Blue = DAPI, green = vimentin. Scale bar: 25 μm.

**Figure 6:**
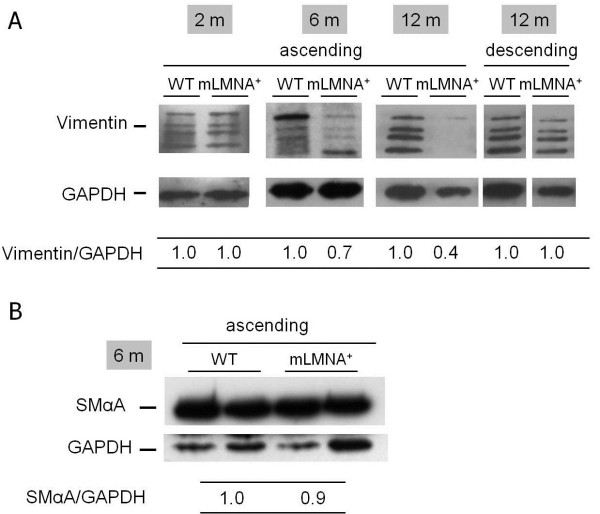
**Western blot analysis of vimentin in the ascending and descending aortas.  (A)** Vimentin expression was investigated by Western blot in 2-, 6- and 12-month-old wild type (WT) and *mLMNA*^*+*^ mice. Blots were performed in triplicate and total vimentin intensity of the four isomer bands was normalized with glyceraldehyde 3-phosphate dehydrogenase (GAPDH) and the ratio indicated. At two months, vimentin was expressed at a similar level in WT and *mLMNA*^*+*^ ascending aortas. However, vimentin expression was reduced by 30% at 6 months and by 60% at 12 months in *mLMNA*^*+*^ ascending aortas. The 12-month descending aortas showed no difference between WT and *mLMNA*^*+*^ mice. **(B)** When the expression bands of SMαA in the ascending aortas of six-month-old WT and *mLMNA*^*+*^ mice were normalized to GAPDH, they were similar (ratio = 1.0:0.9). (*P <0.001).

The cellular pattern of vimentin expression in the ascending aortas was examined in 12-month-old WT and *mLMNA*^*+*^ mice by immunostaining and confocal microscopy. In both groups, the intima was seen as an intact monolayer of vimentin-positive endothelial cells (Figure 5A,C). Vimentin expression was observed in all SMCs in the media of the ascending aortas in WT mice (Figure 5A). Although the surviving SMCs in the aortic media in *mLMNA*^*+*^ mice were vimentin-positive, extensive depletion of these cells resulted in an overall decrease in the level of vimentin (Figure 5C). Thus, visual comparison of vimentin immunostaining of the ascending aortas of these two groups of mice, at 12 months, supported our Western blot findings (Figure 6). To determine the onset of decreased vimentin expression in *mLMNA*^*+*^ mice, we quantified total vimentin levels which included all vimentin isomers in the ascending aortas from 2-, 6- and 12-month-old WT and *mLMNA*^*+*^ mice (Figure 6). Western blot showed no difference at 2 months; however, vimentin in the ascending aortas of *mLMNA*^*+*^ mice was decreased 30% at 6 months and 60% at 12 months compared with the WT group.

Vimentin was expressed in all endothelial cells of the ascending aortas in WT and *mLMNA*^*+*^ mice). Furthermore, consistent with Western blot data, immunostaining of descending aortas at 12 months showed a similar pattern of vimentin expression in all three layers in WT and *mLMNA*^*+*^ mice (Figure 5B,D).

### Effect of shear stress on gene expression

**Figure 7:**
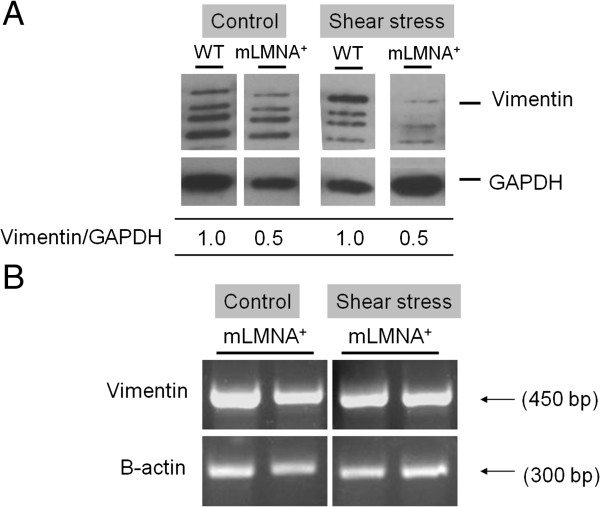
**Western blot and mRNA analysis of vimentin in the descending aortas after artificial shear stress.** Vimentin expression in 12-month-old wild type (WT) and *mLMNA*^*+*^ descending aortas provided control values for a study that involved a 30-minute *ex vivo* exposure to high fluidic shear stress. In triplicate samples no difference was detected between WT and *mLMNA*^*+*^ control samples not exposed to shear stress **(A)**. This image was the same figure as Figure 6A (12 m descending WT and *mLMNA*^*+*^). After exposure to shear stress, a 50% reduction in vimentin expression was demonstrated in *mLMNA*^*+*^ mice compared with WT mice (A). Vimentin mRNA expression in descending aortas of WT and *mLMNA*^*+*^ mice was obtained by RT-PCR analysis after exposure to shear stress **(B)**.

To test our hypothesis that high shear stress contributes to the vasculopathy seen in the media of the ascending aortas of *mLMNA*^*+*^ mice, we applied artificial shear stress at 75 dynes/cm^2^ to the descending aortas of WT and *mLMNA*^*+*^ mice. Western blot analysis (Figure 7A) of descending aortas following exposure to 30 minutes of fluidic shear stress showed a 50% lower vimentin level in *mLMNA*^*+*^ mice compared to the WT group. In an effort to establish whether the decrease in vimentin in *mLMNA*^*+*^ mice resulted from a block at the level of gene transcription, we performed mRNA analysis on descending aortas from WT and *mLMNA*^*+*^ mice subjected to fluidic shear stress and found similar mRNA levels in both groups (Figure 7B), consistent with post-translational degradation of vimentin.

## Discussion

In the *mLMNA*^*+*^ mouse model of progeria, an early indication of vasculopathy consistently occurred as a thickened zone of the media in the ascending aortas, but not in the descending aortas, followed by loss of SMCs in 12-month-old mice. Thus, vasculopathy may develop at a slower rate in the descending aortas where SMCs are exposed to lower hemodynamic stress compared to the ascending aortas. This idea is supported by our finding that when exposed *ex vivo* to high hemodynamic stress, the descending aortas in *mLMNA*^*+*^ mice respond with a decrease in vimentin transcripts and protein compared with WT mice. Our observation of no vasculopathy in the descending aortas at this age differs from an earlier study [9] in which mice were followed for more than 12 months. An explanation for the differences regarding the extent of vasculopathy seen in these two studies is the fact that the earlier study [9] utilized mice that had undergone fewer generational passages [Personal communication, Dr. Francis S. Collins].

Our hypothesis that changes in the media occur in 12-month-old mice in response to high flow pressure generating a mechanical force over the wall of the ascending aortas, is supported by a number of recent papers [11,14]. Phase-contrast MRI and numerical analysis suggest that local wall stress conditions may be the basis for media thickening in the wall of the aortic arches [13]. Additionally, simulated computational models of aortas used to analyze fluid dynamics suggest that hemodynamic stress encountered in the ascending portion of the aortic arches is highest at the level of the medial layer [11,12]. Our calculations, based on MRI measurements, indicate that an 18% decrease in lumen diameter occurs at the origin of the ascending aortas in *mLMNA*^*+*^ mice. This suggests an 80% increase in mechanical stress at this site that is not encountered in WT mice. Thus, repetitive high stress associated with each cardiac systole may be a key factor responsible, over time, for the focal thickening of the media followed by the loss of SMCs in the ascending aortas in *mLMNA*^*+*^ mice.

In an effort to define the molecular basis for SMC depletion in the ascending aortas of *mLMNA*^*+*^ mice, we performed proteomic analyses to identify potential mediators of SMC death. Data obtained from protein profiles of ascending aortas in WT and *mLMNA*^*+*^ mice, and confirmed by Western blot and confocal microscopy, led us to focus on vimentin. This type III intermediate filament, a component of the cytoskeletal system, is known to function along with actin and microtubules to maintain cellular integrity through its role in adhesion, structure and migration [15]. Other attributes of vimentin include its ability to engage in intracellular signaling involved in gene expression [16], and vascular remodeling [17,18]. Another attribute that may be relevant to our study is the role of vimentin in the apoptotic pathway. Several cell types [19-21], including vascular SMCs [22], are reported to undergo apoptosis in response to caspase cleavage of vimentin, whereas caspase-resistant vimentin suppresses apoptosis [23]. Defects in mechanotransduction due to defective SMC vimentin may result in the vasculopathy observed in several of the lamin A disorders. Importantly, some of the functions attributed to vimentin are impaired in HGPS patient fibroblasts subjected to repetitive mechanical strain [24,25].

In an effort to determine whether progerin, the mutant form of lamin A, is involved in the loss of SMCs in the ascending aortas of *mLMNA*^*+*^ mice, we utilized immunohistochemistry and confocal microscopy to monitor its cellular expression. Progerin was detected in all SMCs of the descending aortas. At sites in the medial layer of the ascending aortas of 12-month-old *mLMNA*^*+*^ mice where extracellular matrix replaced the SMCs, the only trace of progerin occurred in rare, individual surviving SMCs. Thus the focal reduction in the number of SMCs in the ascending aortas in *mLMNA*^*+*^ mice formed the basis for the low level of progerin detected by Western blot.

Endothelial cells of the ascending aortas display a unique feature in *mLMNA*^*+*^ mice. They form an intact single cell layer and their survival in the zone of SMC depletion is characterized by a greater than eight-fold level of vimentin compared with cells overlying adjacent regions of media not depleted of SMCs. Implications of a role for vimentin in survival of endothelial cells are supported by studies that demonstrate intermediate filaments confer tolerance to high hemodynamic stress [26,27]. These studies show that vimentin helps endothelial cells to withstand mechanical forces generated by blood flow and report a greater than two- to three-fold level of vimentin in endothelial cells of the aortas, where flow-induced stress is high, compared with the level in endothelium of the vena cava. Furthermore, throughout the aortas there is an increase in vimentin content in a proximal to distal direction [27]. In addition to their function in signal transduction, vimentin filaments in endothelial cells associate with integrins to form focal contacts and help stabilize cell-matrix adhesions. These focal contacts are flow-induced [26]. Data support our view that enhanced expression of vimentin in endothelial cells over the zone of vasculopathy helps to maintain cell viability. Due to their capacity for signal transduction and their ability to form cell-matrix adhesions that offer resistance to high mechanical stress, endothelial cell survival is enhanced in the ascending aortas, regions subjected to high hemodynamic pressure with each systole. In addition to shear stress, the hoop stress could also be considered. Hoop stress influences the vascular wall tension (muscular tension). A simple computational modeling can give more information about how hoop stress influences the endothelial and medial layers of aortas.

A decrease in vimentin, an intermediate filament vital to cellular survival, in the wall of the ascending aortas was detected in six-month-old *mLMNA*^*+*^ mice compared with WT mice, despite no differences in SMαA protein. Because the loss of vimentin preceded the loss of SMαA or any evidence for cell death at this age, we speculated that vimentin loss may contribute to an early stage of the vascular pathology seen in this animal model of HGPS, and that hemodynamic stresses in the ascending aortas may be of mechanistic importance in reduced vimentin expression. To test this concept, we applied mechanical stress to descending aortas that showed little or no signs of vasculopathy at 12 months of age. After a 30-minute exposure to hemodynamic forces equivalent to 75 dynes/cm^2^ there was a significant reduction in vimentin protein expression in the descending aortas of *mLMNA*^*+*^ mice compared with WT mice. We considered vimentin degradation as an additional mechanism for the rapid decline in vimentin expression in these 30-minute experiments. Vimentin is a caspase-3, -6 and -7 substrate that is cleaved at distinct sites producing multiple fragments [21]. Studies utilizing single living cells demonstrated a rapid activation of the caspase cascade leading to apoptosis. Several of these studies demonstrated activation within 5 to 30 minutes after exposure to a variety of stimuli. Within 15 minutes following intestinal epithelial cell detachment, activation of caspase-6 and cleavage of its substrate - poly (ADP-ribose) polymerase - were detected. This was followed within 30 minutes by activation of caspase-3 [28]. In another study, using COS-7 cells, activation of caspase-3 was detected five minutes after the initiation event occurred [29]. Similar findings were reported using single living plant cells. At 30 minutes after exposure to ultraviolet C, activation of caspase-3 was detected based on a decrease in a recombinant fluorescent substrate [30]. Additional studies are required to clarify the potential role of proteolytic enzymes in degradation of vimentin and other mechanotransduction filaments during the onset of vasculopathy in *mLMNA*^*+*^ mice. Furthermore, the potential role of mitochondrial enzymes up-regulated under shear stress condition will be studied.

Extracellular matrix (ECM) is connected to cytoskeleton and could be classified as an ancillary mechanotransduction protein. In our study, collagen VI expression was significantly higher in *mLMNA*^*+*^ ascending aortas compared to WT aortas. Collagen VI provided a structural link between ECM and the cell basement membranes. It translates extracellular mechanical stimuli into intracellular biochemical signals [26]. They are also involved in metabolic changes and muscle phenotypes, and lead to production of altered mechano-sensing, triggering the onset of diseases.

## Conclusions

In conclusion, altered expression of mechanotransduction proteins in SMCs may contribute to the vasculopathy seen in the aortas in *mLMNA*^*+*^ mice. Mechanosensitive genes that participate in the regulation of biochemical pathways and protein expression were recently identified in aortic arch endothelial cells. This raises the possibility that these genes in SMCs may likewise be responsive to altered flow forces in the presence of the nuclear protein, progerin. A more complete understanding of possible mechanistic links involving progerin, vimentin and SMC integrity may have implications for the treatment of HGPS patients.

## Abbreviations

BAC: Bacterial artificial chromosome; ECG: Electrocardiography; ECM: Extracellular matrix; DAPI: 4′, 6-diamidino-2-phenylindole; FITC: Fluorescein isothiocyanate; GAPDH: Glyceraldehyde 3-phosphate dehydrogenase; HGPS: Hutchinson-Gilford Progeria Syndrome; mLMNA: Mutant lamin A gene; MRI: Magnetic resonance imaging; ROI: Region of interest; RT-PCR: Reverse transcriptase-polymerase chain; SMCs: Smooth muscle cells; WT: Wild type.

## Competing interests

The authors declare that they have no competing interests.

## Authors’ contributions

MS designed the experiments, carried out the shear stress experiment, proteomics and Western blotting, analyzed data and drafted the manuscript. HS performed the animal study experiments, collected and analyzed the data, and participated in manuscript drafting. SA performed MRI, assembled and interpreted data, and was involved in drafting the manuscript. RC supervised the design and execution of the experiments, and participated in manuscript writing. DO performed immunostaining and confocal imaging, supervised the experiments, and wrote the manuscript. All the authors read and approved the final manuscript.

## Supplementary Material

Additional file 1 **Microfluidic system for shear stress experiment.** Tubes were connected to the microfluidic chamber and ascending aortas, and the media flowed through the tubes. Click here for file
