# Supplementary figures and images for: Shear stress-induced mechanotransduction protein deregulation and vasculopathy in a mouse model of progeria
Source: Stem Cell Res Ther. 2014 Mar 24;5(2):41. doi: 10.1186/scrt429 (PMC4055145; doi:10.1186/scrt429)

## Slide 1
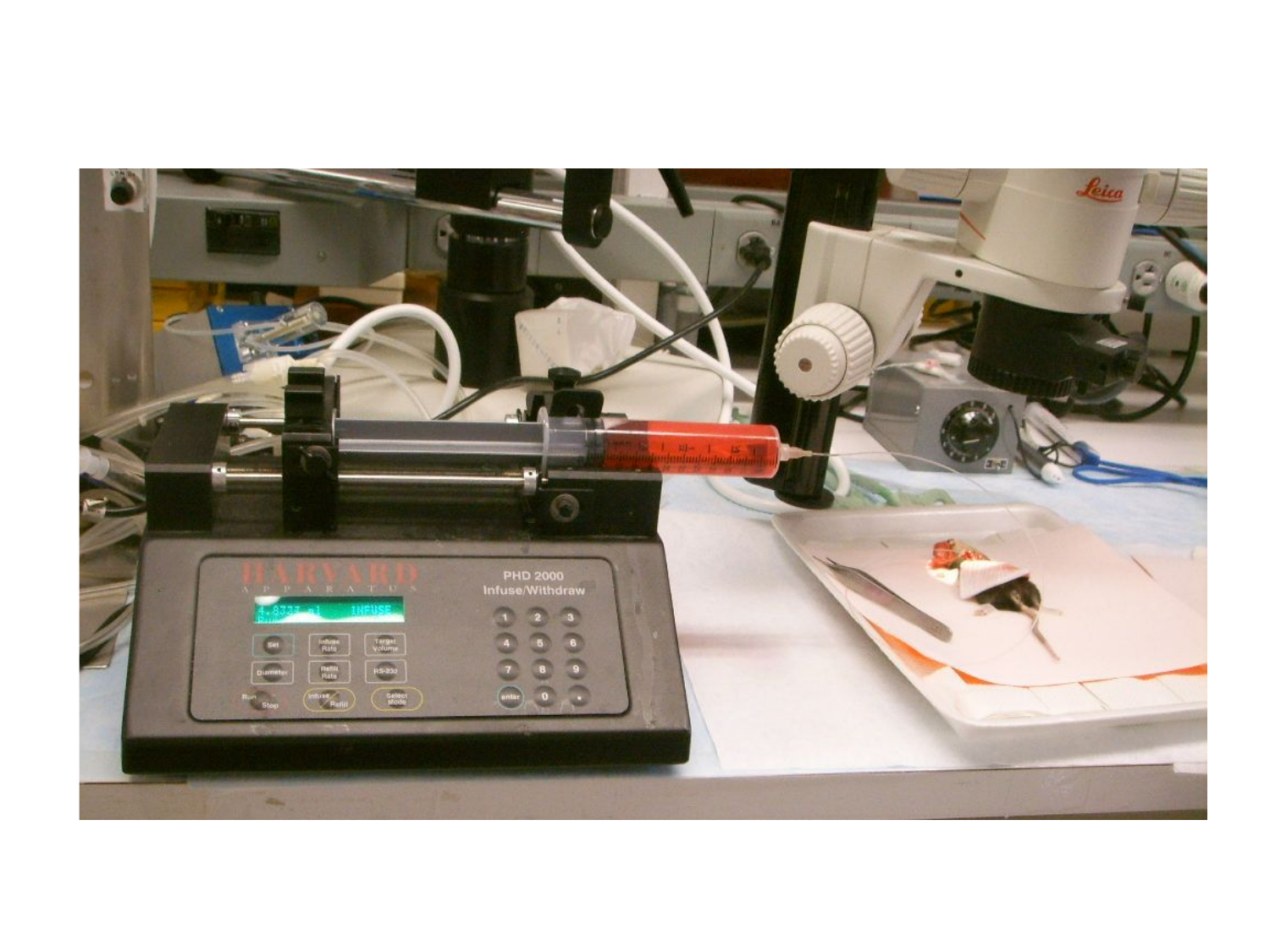

Supplement: Additional file 1 — Microfluidic system for shear stress experiment. Tubes were connected to the microfluidic chamber and ascending aortas, and the media flowed through the tubes. [file scrt429-S1.pptx]
